# Dopamine transporter imaging with [^18^F]FE-PE2I PET and [^123^I]FP-CIT SPECT—a clinical comparison

**DOI:** 10.1186/s13550-018-0450-0

**Published:** 2018-11-15

**Authors:** Susanna Jakobson Mo, Jan Axelsson, Lars Jonasson, Anne Larsson, Mattias J. Ögren, Margareta Ögren, Andrea Varrone, Linda Eriksson, David Bäckström, Sara af Bjerkén, Jan Linder, Katrine Riklund

**Affiliations:** 1 Department of Radiation Sciences, Diagnostic Radiology, Umeå University, Umeå, Sweden; 2 Umeå Center for Functional Brain Imaging, Umeå University, Umeå, Sweden; 3 Department of Radiation Sciences, Radiation Physics, Umeå University, Umeå, Sweden; 4 Department of Integrative Medical Biology, Umeå University, Umeå, Sweden; 5 Department of Clinical Neuroscience, Centre for Psychiatry Research, Karolinska Institutet and Stockholm County Council, Stockholm, Sweden; 6 Department of Pharmacology and Clinical Neuroscience, Umeå University, Umeå, Sweden

**Keywords:** Parkinson’s disease, PET, SPECT, Dopamine transporter (DAT), [^18^F]FE-PE2I

## Abstract

**Background:**

Dopamine transporter (DAT) imaging may be of diagnostic value in patients with clinically suspected parkinsonian disease. The purpose of this study was to compare the diagnostic performance of DAT imaging with positron emission computed tomography (PET), using the recently developed, highly DAT-selective radiopharmaceutical [^18^F]FE-PE2I (FE-PE2I), to the commercially available and frequently used method with [^123^I]FP-CIT (FP-CIT) single-photon emission computed tomography (SPECT) in early-stage idiopathic parkinsonian syndrome (PS).

**Methods:**

Twenty-two patients with a clinical de novo diagnosis of PS and 28 healthy controls (HC) participating in an on-going clinical trial of FE-PE2I were analyzed in this study. Within the trial protocol, participants are clinically reassessed 2 years after inclusion. A commercially available software was used for automatic calculation of FP-CIT-specific uptake ratio (SUR). MRI-based volumes of interest combined with threshold PET segmentation were used for FE-PE2I binding potential relative to non-displaceable binding (BP_ND_) quantification and specific uptake value ratios (SUVR).

**Results:**

PET with FE-PE2I revealed significant differences between patients with a clinical de novo diagnosis of PS and healthy controls in striatal DAT availability (*p* < 0.001), with excellent accuracy of predicting dopaminergic deficit in early-stage PS. The effect sizes were calculated for FE-PE2I BP_ND_ (Glass’s Δ = 2.95), FE-PE2I SUVR (Glass’s Δ = 2.57), and FP-CIT SUR (Glass’s Δ = 2.29). The intraclass correlation (ICC) between FE-PE2I BP_ND_ FP-CIT SUR was high in the caudate (ICC = 0.923), putamen (ICC = 0.922), and striatum (ICC = 0.946), *p* < 0.001. Five of the 22 patients displayed preserved striatal DAT availability in the striatum with both methods. At follow-up, a non-PS clinical diagnosis was confirmed in three of these, while one was clinically diagnosed with corticobasal syndrome. In these patients, FE-PE2I binding was also normal in the substantia nigra (SN), while significantly reduced in the remaining patients. FE-PE2I measurement of the mean DAT availability in the putamen was strongly correlated with BP_ND_ in the SN (*R* = 0.816, *p* < 0.001). Olfaction and mean putamen DAT availability was correlated using both FE-PE2I BP_ND_ and FP-CIT SUR (*R* ≥ 0.616, *p* < 0.001).

**Conclusion:**

DAT imaging with FE-PE2I PET yields excellent basic diagnostic differentiation in early-stage PS, at least as good as FP-CIT SPECT.

## Background

Dopamine transporter (DAT) imaging is a key supporting diagnostic examination to distinguish patients with idiopathic parkinsonism (PS) (e.g., Parkinson’s disease, multisystem atrophy, progressive supranuclear palsy, and Lewy body dementia) from patients with Parkinson-like symptoms with preserved dopaminergic function. DAT imaging with the commercially available radiopharmaceutical [^123^I]FP-CIT ([^123^I]-ioflupane, DaTSCAN™, GE Healthcare, B.V Eindhoven, NL) SPECT (FP-CIT) is an established method, widely used in both clinical practice and research projects [1]. In recent years, a novel radiopharmaceutical for DAT imaging, [^18^F]FE-PE2I ([^18^F]-(E)-*N*-(3-iodoprop-2-enyl)-2β-carbofluoroethoxy-3β-(4′-methyl-phenyl) nortropane) for PET (FE-PE2I), has emerged as a promising alternative, allowing a more selective and detailed DAT visualization and quantification [2, 3]. PET imaging with FE-PE2I permits faster patient throughput due to faster kinetics, allowing for reduced time between injection and imaging, and a shorter static imaging protocol [2, 4]. There is no need for pre- and post-scan administration of thyroid protecting agents when using [^18^F], and the effective dose is comparable to FP-CIT [5]. Moreover, the spatial and temporal resolution in PET is superior compared to SPECT, and in contrast to conventional DAT SPECT imaging, in vivo visualization and quantification of DAT in extra-striatal regions, such as the substantia nigra (SN) [6, 7], is feasible with FE-PE2I. This is due to the high affinity (*K*_D_ = 12 nM) [8] and superior selectivity for DAT over the serotonin transporter (SERT), a ratio which is found to be 29.4 for PE2I [9] and 2.78 for FP-CIT [10]. The binding of FP-CIT in the midbrain is to the serotonin transporter, not to the dopamine transporter, whereas FE-PE2I is indeed selective for the DAT. The quantification of the DAT binding in the substantia nigra indicates the availability of the DAT on the cell bodies of the dopaminergic neurons. Therefore, FE-PE2I is a potentially attractive, high-quality PET imaging alternative tool for visualization of presynaptic dopamine integrity both in the striatum and in the SN in research and clinical practice. Despite the potential advantages of FE-PE2I compared to FP-CIT, these two ligands have previously not been directly compared in patients. The aim of this study was to compare the basic diagnostic performance of FE-PE2I and FP-CIT in patients with newly onset parkinsonism, fulfilling the step 1 clinical criteria for an idiopathic parkinsonian syndrome at first visit, and healthy control subjects (HC). In clinical practice, the visual interpretation of DAT imaging is very important; however, quantitative measurements are often also used, whereas in research settings the quantitative assessment is essential. Here, the primary objective was to measure the effect size and accuracy of these two DAT imaging methods in separating early-stage PS patients from control subjects, using clinically relevant quantitative image evaluation methods. The secondary objectives were to measure the intraclass correlation coefficient between FE-PE2I and FP-CIT outcome measures, to measure the correlation between DAT availability and clinical measures in PS patients, and to examine the usefulness of the quantification of DAT availability in the SN.

## Methods

### Patients and healthy participants

Patients within the age range of 45–80 years with newly onset clinical symptoms of suspected PS, who were referred for a first-visit specialist evaluation at the neurological department at Umeå University Hospital, were eligible for participating in the research project, which is an on-going non-profit clinical trial of FE-PE2I. The inclusion period was between November 2015 and May 2018. Patients were included if not previously diagnosed with a neurological, neurodegenerative, neurovascular, or psychiatric disorder and if fulfilling the UK Parkinson’s Disease Society Brain Bank (UKPDBB) step 1 clinical diagnostic criteria for a parkinsonian syndrome [11]. Exclusion criteria were also severe physical illness, claustrophobia, or other contraindications against MRI, pregnancy, or nursing.

A reference group of 30 HC was recruited by advertisements in the local newspaper. Men and women within the age range of 60–80 years were eligible if in general good health and without any of the exclusion criteria listed for the patients. All healthy volunteers were investigated with the same protocol as the patients. A protocol-based clinical follow-up 2 years after inclusion for diagnostic re-evaluation is scheduled for all participants.

Sixteen of the HC in this study have been clinically confirmed still healthy at the clinical follow-up visit; however, only eight patients have been clinically reassessed up to now. Of these, one patient fulfil the clinical criteria for corticobasal syndrome (CBS), four patients have Parkinson’s disease (PD), and three patients do not fulfil the criteria for any of the neurodegenerative parkinsonian syndromes, but have been diagnosed with essential tremor, vascular parkinsonism, and undetermined tremor, respectively.

The multi-modal methodology for PET image analysis used in this study failed in two HC, and they were consequently excluded from analysis in this particular study.

The study group in this paper comprises the first included 22 patients (10 women and 12 men), mean age 68.8 ± 8.0 years, and 28 HC (13 women and 15 men), mean age 70.1 ± 4.6 years. The basic clinical features of the patients and HC in the study are presented in Table 1.

**Table 1:** Basic data

		HC *n* = 28	PS *n* = 22	*p*
Age	Mean (SD)	69.8 (4.4)	68.8 (8.0)	*0.950*
Body weight, kg	Mean (SD)	73.4 (10.9)	76.4 (12.8)	*0.226*
FE-PE2I dosage, MBq	Mean (SD)	176.0 (21.5)	165.9 (14.0)	*0.120*
Olfactory function^a^	Normal/pathologic	25/3	4/14	*< 0.001*
Sniff test, points^a^	Mean (SD)	10.1 (1.1)	6.5 (2.8)	*< 0.001*
UPDRS-III	Median (min-max)	1.0 (0–5)	22.5 (9–33)	*< 0.001*
Symptoms’ duration, years	Mean (SD)	NA	1.1 (0.8)	–
H&Y	Median (min-max)	NA	2 (1–4)	–

*HC* healthy controls; *PS* parkinsonian syndrome; *UPDRS-III* Unified Parkinson’s Disease Rating Scale, motor part; *H&Y* Hoehn and Yahr disease stage score; *NA* not applicable

^a^Olfactory testing data available for 18 patients

### Clinical evaluation and imaging protocol

Patients and HC were evaluated clinically by a neurologist with special interest in movement disorders (JL, SaB, LE, and DB) and by a research nurse specialized in movement disorders. Clinical testing included motor function evaluation according to the third section in the Unified Parkinson’s Disease Rating Scale (UPDRS-III) and Hoehn and Yahr disease severity score (H&Y). Olfactory function was assessed with the Brief Smell Identification Test™ (B-SIT, Sensonics International).

After inclusion and clinical evaluation, patients and HC were examined with FP-CIT and a dynamic FE-PE2I separated by 5–21 days. For the patients, the clinical diagnosis at baseline was made before imaging and hence unbiased by the results of any imaging modality, and DAT imaging was always done before commencement of any medical treatment. The follow-up clinical diagnosis was based on established clinical criteria, in off medicated state.

### Image acquisition

#### PET/CT

FE-PE2I was synthesized on-site at Umeå University Hospital as previously described [5]. PET/CT imaging was performed with a Discovery 690 PET/CT (GE Healthcare, Milwaukee, WI).

**Fig. 1:**
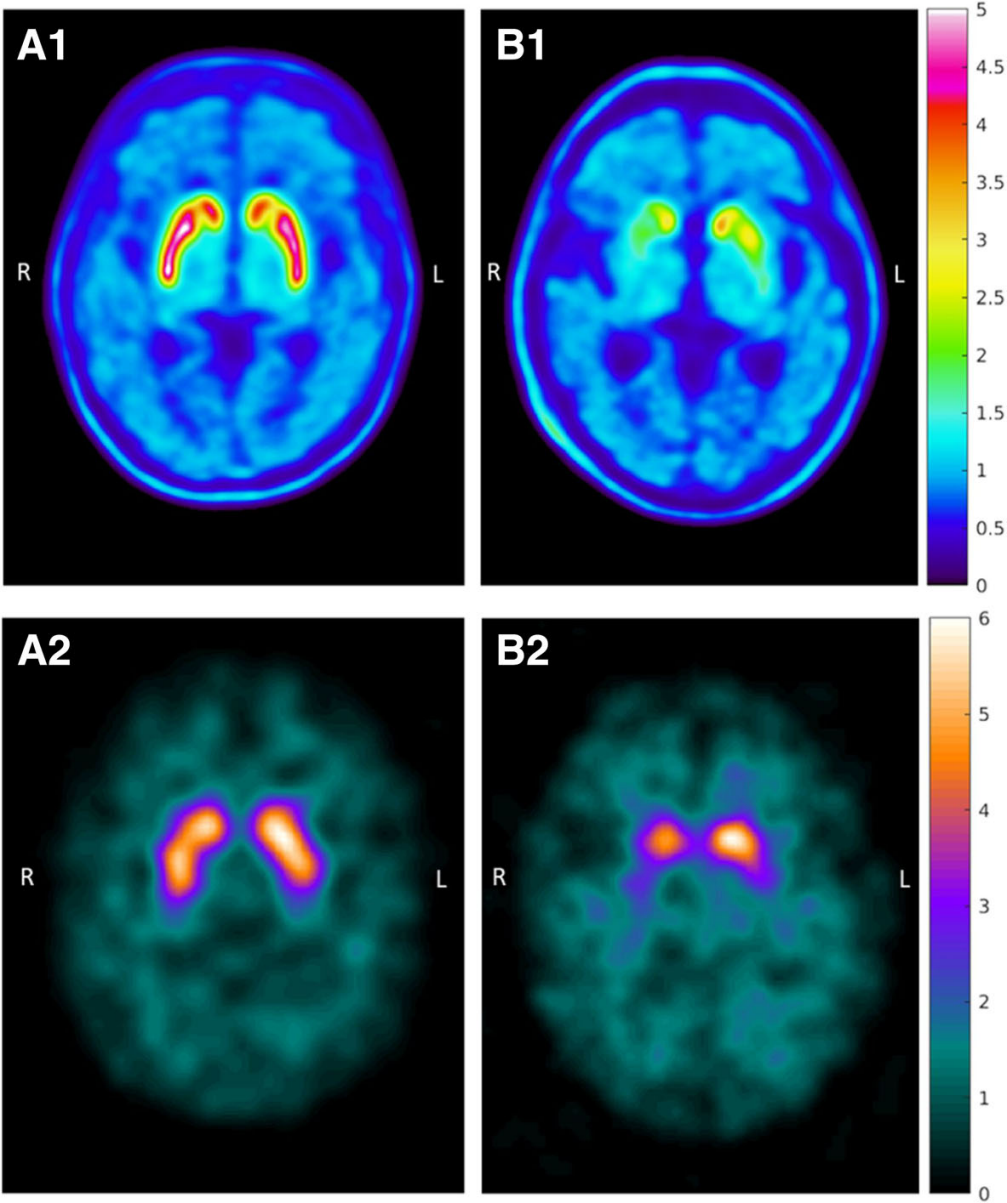
Examples of [^18^F]FE-PE2I PET and [^123^I]FP-CIT SPECT images in the same individuals. Representative images of FE-PE2I PET (parametric DVR images) and FP-CIT SPECT (SUR images) of the same two subjects. Healthy control examined **A1** with FE-PE2I and **A2** with FP-CIT. Patient with parkinsonian syndrome since 6 months examined **B1** with FE-PE2I PET and **B2** with FP-CIT SPECT. *PET image scale*: DVR (= BP_ND_ + 1). *SPECT image scale*: activity uptake in striatum divided by cerebellum activity

Following a low-dose CT (120 kV, 10 mA, 0.8-s rotation), a 75-min dynamic PET acquisition (9 × 20 s, 3 × 60 s, 5 × 180 s, 9 × 360 s) commenced at bolus intravenous injection of FE-PE2I. Target dose was 2.86 MBq/kg, limited to 200 MBq above 70 kg (Table 1**)**. PET images were reconstructed with the SharpIR iterative algorithm (6 iterations, 24 subsets, 3.0 mm post-filter) employing time-of-flight, decay, attenuation, and scatter correction [12, 13]. The full width at half maximum resolution for this reconstruction is measured to 3.2 mm radially in the slice plane and 4.7 mm in the axial direction. An individually fitted thermoplastic mask restricted head movement. No serious adverse effects or adverse events related to FE-PE2I PET/CT imaging have occurred. Examples of a representative FE-PE2I image in one healthy subject and one PS patient participating in the study are displayed in Fig. 1. The corresponding FP-CIT images of the same individuals are also shown.

#### SPECT/CT

FP-CIT was purchased from GE Healthcare (B.V Eindhoven, NL). Dosage and scanning procedures of FP-CIT followed the manufacturer’s recommendations. Imaging was acquired with a hybrid all-purpose dual-head SPECT/CT scanner, the Discovery NM/CT 670 Pro (GE Healthcare, Milwaukee, WI, USA) equipped with low-energy high-resolution (LEHR) collimators. Imaging was commenced 3 h after an intravenous bolus injection of 185 MBq of the radiopharmaceutical. The SPECT projections were acquired for 30 s per projection in a 360° stepwise rotation of detectors, with a rotation radius of 15 cm. Data was collected in 128 × 128 matrices with a zoom factor of 1.5, rendering a pixel size of 2.94 × 2.94 mm. Image reconstruction was done with OSEM (8 iterations, 6 subsets) and post-filtered with a Butterworth filter (critical frequency 0.45 cm^−1^, order 8). The triple energy window technique [14] was applied for scatter correction, and data derived from an ultra-low-dose CT scan that was acquired in the same session was used for non-uniform attenuation correction. The full-width-at-half-maximum resolution for this reconstruction is measured to 12.5 mm, isotropically. The total scanning time of the protocol, including low-dose CT, is 35 min. Representative examples of FP-CIT images are shown in Fig. 1.

#### MRI

A 3-T MRI (Discovery MR750, GE Medical Systems Milwaukee, WI, USA) was used to acquire a high-resolution 3D fast spoiled gradient echo T1-weighted sequence (180 slices, 1 mm thickness, TR = 8.2 ms, TE = 3.2 ms, flip angle 12°, field of view 25 × 25 cm) and a susceptibility-weighted angiography (SWAN) T2*-sequence (2 mm thickness, TR-minimum = 24.3 ms, TE = 24.4 ms, flip angle 10°, field of view 22 × 22 cm).

### Image analysis

#### Preprocessing

PET image data was corrected for movement using the Linear Image Registration Tool (FLIRT) (https://fsl.fmrib.ox.ac.uk/), using the average of frames 18–20 as a reference volume. FLIRT was used for the rigid-body transformation between the PET and T1 image volumes. The average of frames 5 to 10 was used for coregistration. Freesurfer version 6.0.0 (http://surfer.nmr.mgh.harvard.edu/) [15] was used for automatic segmentation of the T1 images, creating caudate and putamen volumes of interest (VOIs), and the outline of the cerebellar reference region in each individual participant. The VOIs were transformed to the PET native images, where all analyses were performed.

#### PET quantification

**Fig. 2:**
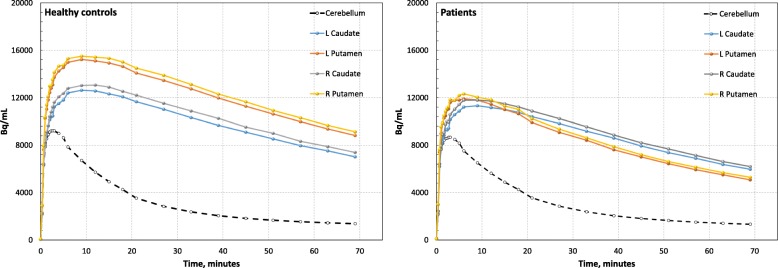
[^18^F]FE-PE2I time-activity curves. [^18^F]FE-PE2I time-activity curves extracted from volume of interests in the native PET image matrix. Left: pooled data acquired in the healthy controls. Right: pooled data acquired in the patients fulfilling criteria for parkinsonian syndrome at inclusion

**Fig. 3:**
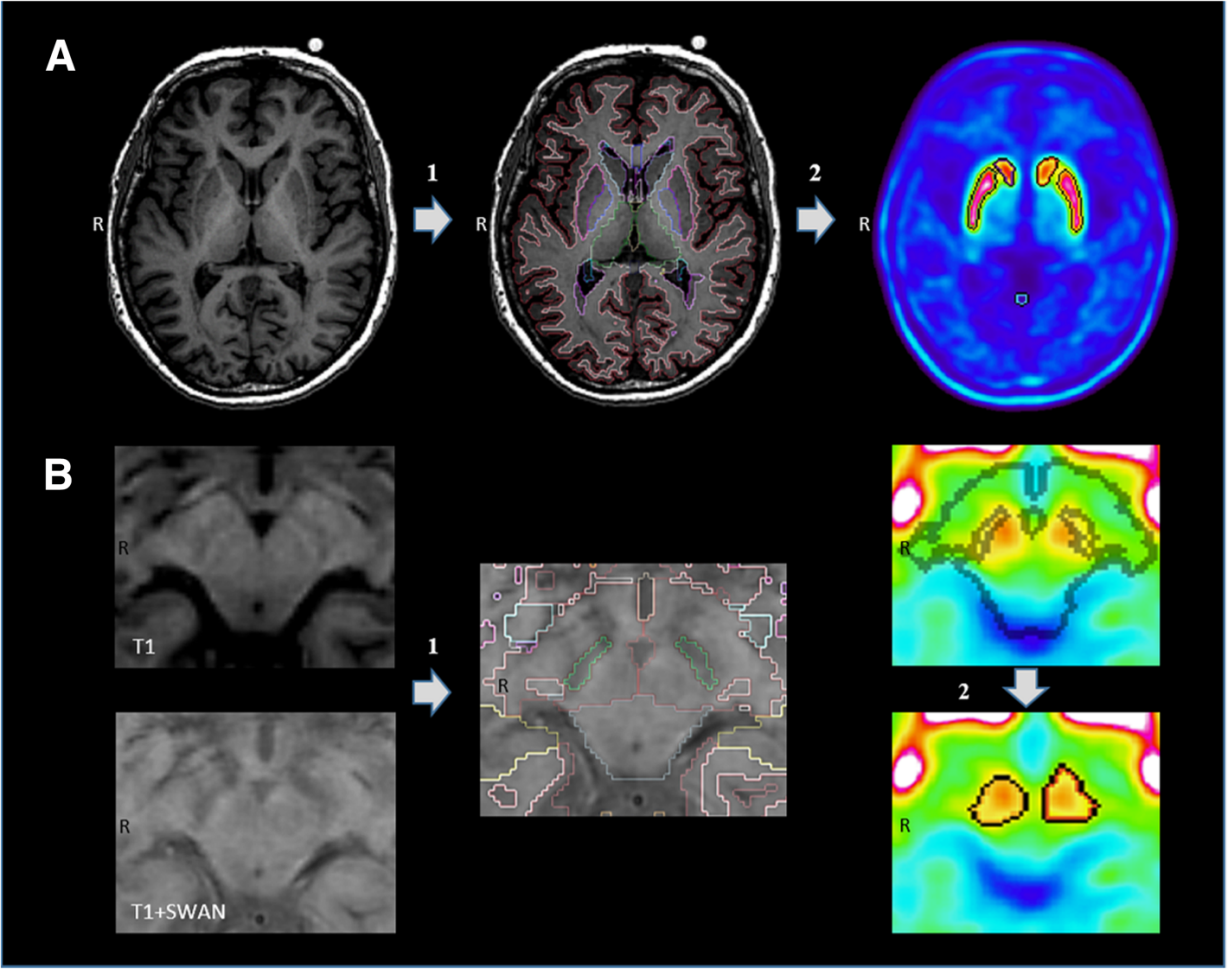
Illustration of the procedure for PET image analysis. **a** Measurement of the FE-PE2I activity in the caudate and the putamen: In the first step (1), T1-weighed MRI data was automatically segmented using Freesurfer, delineating the structural borders of the caudate and putamen in each hemisphere. In the second step (2), delineation of the PET-data was done, restricted by the limits of the segmented VOIs created in the first step. New PET-based VOIs were delineated for the caudate and putamen, defined as the voxels with the highest activity within a volume of 2.0 and 2.5 mL respectively. This was done to adjust for imprecise Freesurfer MRI segmentation, due to periventricular white matter irregularities, and to minimize partial volume effects in the PET image data. **b** Measurement of the FE-PE2I activity in the SN. In the first step (1), manual segmentation was anatomically guided by the hypo-intense area in the midbrain corresponding to SN on merged SWAN and T1-weighted MRI transversal slices. Since the MRI-guided VOIs did not entirely overlap the area of the observed FE-PE2I binding in the midbrain, in the second step (2), these segmented VOIs were used to guide an automatic thresholding algorithm to determine PET-based midbrain VOIs. FE-PE2I, [^18^F]FE-PE2I PET; MRI, magnetic resonance imaging; VOI, volume of interest; SN, substantia nigra; SWAN, susceptibility-weighted angiography

MRI anatomical VOIs were found to be overestimated in size. To resolve this problem, PET activity values were used on the MR-based anatomical VOIs to limit the PET-active volume in a reproducible manner. VOI-based analysis was then performed to extract specific uptake value ratios (SUVR), i.e., uptake/reference ratios and non-displaceable binding (*BP*_ND_) both using the entire cerebellum as reference. The average time-activity curves for patients and HC are displayed in Fig. 2. The PET quantification is schematically illustrated in Fig. 3. A more detailed description follows below.

The PET delineation algorithms, BP_ND_ calculations, and VOI analyses were coded in Matlab R2017b (Mathworks Inc., Natick, MA, USA), employing SPM 8 to read Nifti files (http://www.fil.ion.ucl.ac.uk/spm/).

The VOIs were delineated according to the following steps:

1. MR-based segmentation

The cerebellum, putamen, and caudate nucleus were automatically segmented with Freesurfer in each participant. The SN is not automatically segmented by Freesurfer. Instead, the VOIs for the right and left SN were drawn on the segmentation map in Freeview 2.0 (independently by authors SJM and SaB), guided by anatomical landmarks provided by T1-weighted transversal MRI images overlaying the SWAN images (in which the SN pars reticulata has a low signal) for better contrast. However, the exact anatomical position of the SN pars compacta, where the density of dopaminergic neurons and the DAT availability is expected to be highest, was not possible to delineate with adequate accuracy on available MRI sequences. In fact, the binding of FE-PE2I was observed to be located in the area medial to the MRI-guided VOIs, in the space between (and somewhat overlapping) the hypo-intense part of the SN and the red nucleus, and this area possibly corresponds to the location of the SN pars compacta [16].

2) PET segmentation, based on activity concentration

Due to overestimation of MR-segmented VOI volumes in the putamen and caudate nucleus, and the mismatch between the manually segmented VOI for the SN and the peak activity in the area, PET-guided automatic segmentation was performed for the putamen, caudate, and SN. The following approaches were used, which also adapted to differences in contrast and how the areas were affected by the PET resolution:

*The putamen* was delineated based on the PET activity within the borders of the MR-segmented VOI for the putamen by finding the 799 highest pixels (volume *V*_putamen_ = 2.5 mL).

*The caudate nucleus* was delineated based on the PET activity within the borders of the MR-segmented VOI for the caudate nucleus, by finding the 649 highest pixels (*V*_caudate_ = 2.0 mL).

*In order to cover the peak activity in the SN*, the activity was thresholded at level *A*_th_ within a restricted search volume (*x*, *y*, *z* = 17, 13, 10 mm) positioned at the (mass center) midpoint of the manually MR-segmented left and right SN, respectively. The threshold level *A*_th_ was defined as:

$$ {A}_{\mathrm{th}}=0.5\left({A}_{\mathrm{max}}-{A}_{\mathrm{ref}}\right)+{A}_{\mathrm{ref}} $$

where *A*_max_ is the highest voxel value and *A*_ref_ is the reference region average value.

For the calculation of *BP*_ND_ on the dynamic PET data, the activity-based delineation was performed on an image summed between 27 and 75 min. For the calculations of SUVR, simulated static PET images were created by using the data collected between 51 and 75 min after injection, and the activity-based delineation was performed on these images, created to mimic a static PET scanning protocol, which could be achieved in a clinical scenario.

3) PET quantification

### *BP*_ND_ calculations

The* BP*_ND_, defined in [17] of FE-PE2I, was calculated for the activity curves from the VOIs described in step (2), applying the reference Logan method without the *k*_2_ term (Eq. 7) [18]. Using the variant without the *k*_2_ term was justified by sampling a few subjects, finding that the *BP*_ND_ values varied by less than 5% compared to Reference Logan with *k*_2_ precalculated from the simplified reference tissue model [19]. The entire cerebellum was used as reference region.

The distribution volume ratio (DVR) was calculated by linear regression for times 27 to 75 min, where the Logan curve was visually inspected to be linear. The binding potential was calculated as BP_ND_ = DVR − 1.

Whole striatal BP_ND_ was calculated as a weighted sum of the caudate nucleus and putamen using the equation:

$$ \frac{{\mathrm{BP}}_{\mathrm{ND}}^{\mathrm{caudate}}\times {V}_{\mathrm{caudate}}+{\mathrm{BP}}_{\mathrm{ND}}^{\mathrm{putamen}}\times {V}_{\mathrm{putamen}}}{V_{\mathrm{caudate}}+{V}_{\mathrm{putamen}}} $$

#### SUVR calculations

The SUVR for the putamen, caudate nucleus, and SN were calculated by dividing the VOI value in the respective regions by the uptake value in the cerebellum VOI. The SUVR value for whole striatum was calculated in analogy to the whole striatal *BP*_ND_, as described above.

**Fig. 4:**
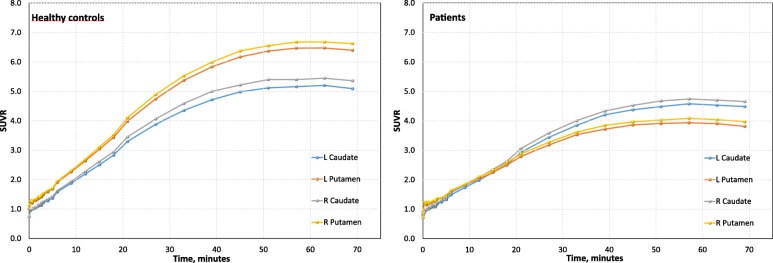
SUVR as a function of time. SUVR (activity/cerebellum ratio) as a function of time for FE-PE2I in healthy controls (left) and PS patients (right). The SUVR reaches an equilibrium 50 min post injection

In the clinical setting, a static imaging protocol would be more feasible than a long dynamic scan; therefore, SUVR would be a suitable semi-quantitative outcome measure. A previous PET study with FE-PE2I has shown that the calculation of SUVR at the time interval around the peak equilibrium provides the best agreement with the estimation of *BP*_ND_ [4]. However, during the dynamic scan, SUVR of FE-PE2I reached a plateau in the 50–75 min time interval (Fig. 4). This condition of pseudoequilibrium is achieved for FP-CIT within 3 to 6 h, which is typically the time interval at which the semi-quantitative specific uptake ratio (SUR) is measured in a static SPECT acquisition. Therefore, to obtain a semi-quantitative measure of SUVR with FE-PE2I in a comparable way as FP-CIT, a static scan within the time interval 50 to 75 min post injection was used. Static images were created by summing the dynamic scans between 51 and 75 min. VOIs were created on this summed image, as described in step (2) above.

#### SPECT semi-quantification

The commercially available software DaTQUANT™ (GE Healthcare, Little Chalfont, UK), installed on a Xeleris 3.1 Workstation, was used for automatic FP-CIT semi-quantitative image analysis. After SPECT reconstruction, the transaxial slices were used as input to the software, which uses an automatic VOI-based semi-quantitative evaluation of the image data and compares it to an in-built set of FP-CIT reference data for statistical analysis. Pre-defined template VOIs are automatically positioned in the striatum, putamen, and caudate in each hemisphere, and in the occipital reference region. The program calculates the semi-quantitative SUR as the difference between the mean counts in each VOI and the mean counts in the background, divided by the mean counts in the background. Putamen/caudate ratios and left/right asymmetry are also presented if desired. The built-in normal database, providing normal age- and sex-stratified reference values, was not used in this study, since our SPECT image reconstruction protocol differed slightly from that used for the normal material of the software. Instead, the HC in the present study provide suitable reference values for the patients’ image data.

### Statistical analysis

The statistical analyses were made in SPSS (IBM SPSS Statistics for Windows, Version 25.0).

The Mann-Whitney *U* test was used for mean differences between groups, applying the exact significance test option. Effect size (ES) calculation was based on the means and standard deviations of left-right averaged PET and SPECT data, using the formula for Glass^’^s Δ = (Mean_1_ − Mean_2_)/SD_HC_, where Mean_1_ = HC and Mean_2_ = patients and SD_HC_ = the standard deviation of the mean in the HC group. Bivariate correlations were tested using Pearson’s correlation coefficient or Spearman’s rho with the two-tailed significance test option. Reliability analysis was done in SPSS using the intraclass correlation coefficient (ICC) to index the reliability of DAT availability measured with SPECT and PET, reflecting both the correlation and measurement agreement between the two methods. The ICC for consistency (cICC) between the methods was calculated, using the two-way mixed-effects model. The discriminative diagnostic capacity of FE-PE2I and FP-CIT was analyzed graphically with receiver operating characteristics (ROC) curves, used as an index of diagnostic accuracy.

For simplicity, left and right striatal compartments were averaged for plots and statistical analysis where applicable. In patients with lateralized symptoms according to the UPDRS-III and clinical examination, the putamen and caudate contralateral to the most affected body half was identified; the hemisphere with the lowest DAT availability was used if symmetrical symptoms’ distribution.

Numbers are expressed as mean ± 1SD if not stated otherwise. The level of significance was set to *p* < 0.05.

## Results

### DAT availability in healthy controls and patients

There were no statistically significant sex or age differences in DAT availability or age in the control group. Analysis of the entire group of HC showed a significant age correlation with FP-CIT SUR, FE-PE2I BP_ND_, and SUVR in the caudate, putamen, and whole striatum (Table 2). However, this age-related correlation was driven by the female HC, since no significant age correlation with DAT activity was evident in men. In the group of patients with pathologic DAT availability, there was also a significant correlation between age and FP-CIT SUR and between age and FE-PE2I BP_ND_ (except for the SN).

**Table 2:** Age correlation with DAT activity, measured with FP-CIT SPECT and FE-PE2I PET

	HC, *n* = 28			PS, *n* = 22		
	All *R*	All *p*	Female/male *R*	All *R*	All *p*	Low DAT/normal DAT *R*
Caudate						
FP-CIT, SUR	− 0.496	*0.007*	− 0.599*/− 0.360^†^	− 0.354	*0.107*	− 0.565*/− 0.396 ^†^
FE-PE2I, *BP*_ND_	− 0.577	*0.001*	− 0.803**/− 0.402^†^	−0.569	*0.006*	− 0.679**/− 0.965**
FE-PE2I, SUVR	− 0.502	*0.007*	− 0.796**/− 0.295^†^	− 0.546	*0.009*	− 0.649**/− 0.970**
Putamen						
FP-CIT, SUR	− 0.400	*0.035*	− 0.543^†^/− 0.161^†^	− 0.207	*0.355*	− 0.612**/− 0.544 ^†^
FE-PE2I, *BP*_ND_	− 0.540	*0.003*	− 0.697**/− 0.395^†^	− 0.153	*0.497*	*− 0.475* ^†^ */− 0.564* ^†^
FE-PE2I, SUVR	− 0.430	*0.022*	− 0.556*/− 0.287^†^	− 0.150	*0.505*	*− 0.433* ^†^ */− 0.698* ^†^
Striatum						
FP-CIT, SUR	− 0.449	*0.016*	− 0.567*/− 0.280^†^	− 0.267	*0.230*	− 0.602*/− 0.496 ^†^
FE-PE2I, *BP*_ND_	− 0.588	*0.001*	− 0.780**/− 0.424^†^	− 0.328	*0.136*	− 0.610**/− 0.832 ^†^
FE-PE2I, SUVR	− 0.480	*0.010*	− 0.675*/− 0.302^†^	− 0.316	*0.153*	− 0.568*/− 0.916*
SN						
FE-PE2I, *BP*_ND_	0.071	*0.719*	*0.246* ^†^ */− 0.117* ^†^	− 0.175	*0.436*	*− 0.333* ^†^ */− 0.221* ^†^
FE-PE2I, SUVR	0.058	*0.768*	*0.199*^†^/− 0.460^†^	− 0.152	*0.500*	*− 0.273* ^†^ */− 0.139* ^†^

Correlations are calculated for mean DAT availability values in the respective compartments (averaged between the left and right hemispheres). Patients with low DAT *n* = 17, patients with normal DAT *n* = 5

*SUR* specific uptake ratio, *BP*_ND_ binding potential relative to non-displaceable binding, *SUVR* specific uptake value ratio, *SN* substantia nigra, *HC* healthy controls, *PS* parkinsonian syndrome

*Correlation is significant at the 0.05 level (two-tailed)

**Correlation is significant at the 0.01 level (two-tailed)

^†^Correlation is not significant (two-tailed)

All three methods identified five patients with baseline clinical PS but normal striatal DAT activity. One of these fulfilled clinical criteria for CBS at follow-up 2 years after scanning, and three had essential tremor, vascular parkinsonism, or undetermined tremor. The fifth patient with normal DAT has not yet been clinically re-evaluated.

Both FE-PE2I and FP-CIT revealed a significant difference in DAT availability between PS and HC, and the mean averaged DAT availability in the right and left caudate, putamen, and striatum is shown in Table 3.

**Table 3:** Mean FP-CIT SPECT and FE-PE2I PET binding in HC and patients

	HC, *n* = 28		PS, *n* = 22		Sig.	Effect size
	Mean (SD)		Mean (SD)		*p*	Glass’s Δ
FP-CIT, SUR						
Caudate	3.12	(0.46)	2.35	(0.79)	*< 0.001*	1.67
Putamen	2.62	(0.47)	1.43	(0.68)	*< 0.001*	2.54
Striatum	2.80	(0.45)	1.77	(0.69)	*< 0.001*	2.29
FE-PE2I, BP_ND_						
Caudate	2.93	(0.46)	2.29	(0.80)	*0.004*	1.40
Putamen	3.78	(0.46)	1.98	(1.07)	*< 0.001*	3.90
Striatum	3.40	(0.43)	2.12	(0.90)	*< 0.001*	2.95
SN	0.72	(0.07)	0.60	(0.10)	*< 0.001*	1.62
FE-PE2I, SUVR						
Caudate	5.54	(0.75)	4.61	(1.28)	*0.006*	1.24
Putamen	6.75	(0.83)	3.97	(1.71)	*< 0.001*	3.36
Striatum	6.21	(0.76)	4.26	(1.44)	*< 0.001*	2.57
SN	2.08	(0.12)	1.88	(0.18)	*< 0.001*	1.67

Measures of DAT availability in the caudate, putamen, striatum, and SN. Mean = averaged left and right side

*SUR* specific uptake ratio, *BP*_ND_ binding potential relative to non-displaceable binding, *SUVR* specific uptake value ratio, *HC* healthy controls, *PS* parkinsonian syndrome, *SN* substantia nigra

### Effect size and receiver operating characteristics

While the effect size (ES) was high for both methods, as shown in Table 3, especially in the putamen, FE-PE2I had slightly higher ES than FP-CIT.

The ROC curve analysis, taking all the patients and HC into account for averaged left and right putamen DAT availability measured with dynamic FE-PE2I BP_ND_ without correction for age, showed an area under the curve (AUC) of 0.903 (95% CI 0.809–0.997), for static FE-PE2I SUVR AUC = 0.886 (95% CI 0.786–0.986), and for FP-CIT SUR the AUC was 0.891 (95% CI 0.786–0.994). Adjustment for age yielded similar numbers, i.e., for FE-PE2I BP_ND_, the AUC was 0.893 (95% CI 0.787–0.998); for FE-PE2I SUVR, the AUC was 0.890 (95% CI 0.789–0.990); and for FP-CIT SUR, the AUC was 0.899 (95% CI 0.8023–0.996). Thus, both FE-PE2I and FP-CIT have excellent discriminative properties in the differentiation between patients with dopaminergic deficit and healthy controls.

### Reliability

**Fig. 5:**
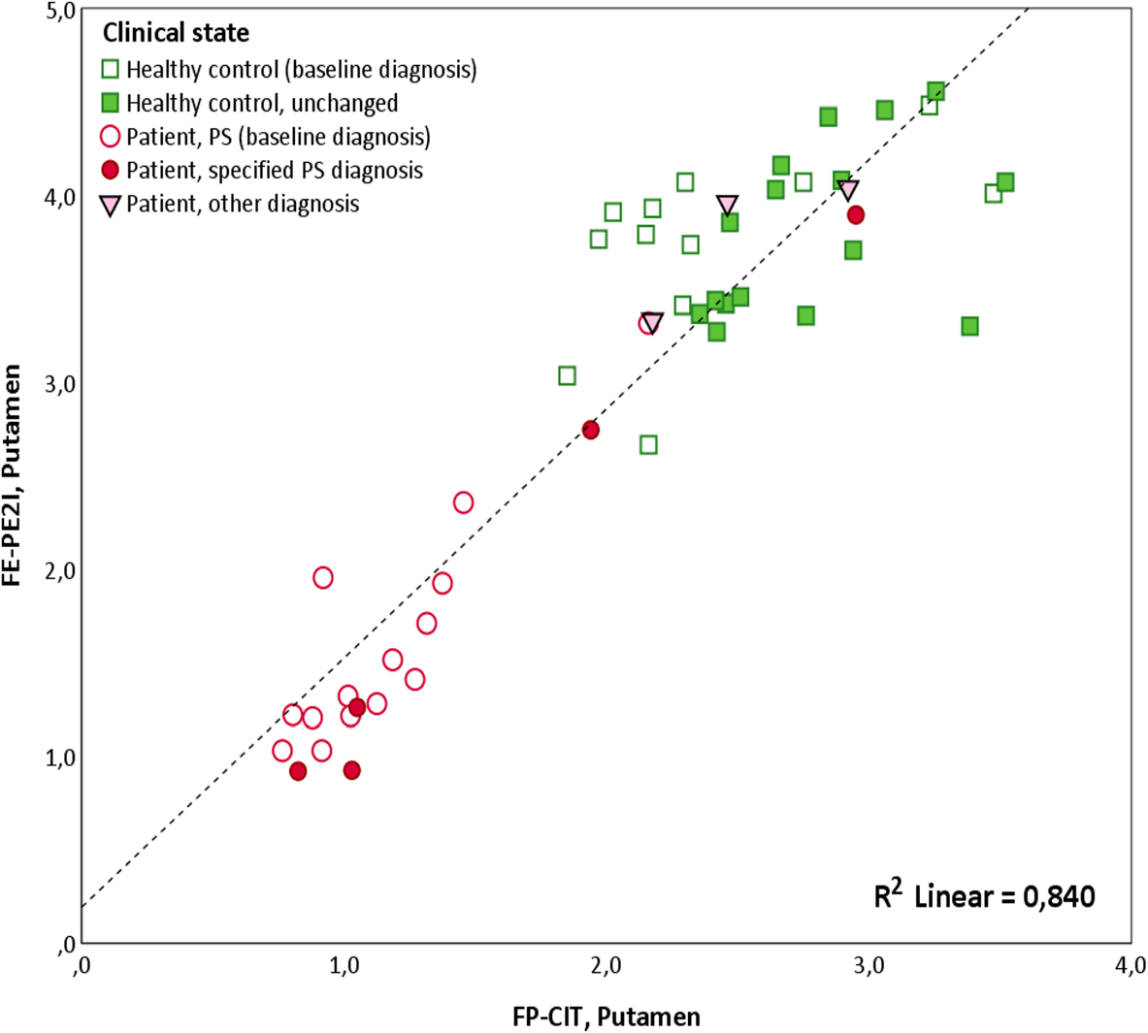
DAT availability in the putamen measured with FE-PE2I versus FP-CIT. Scatterplot of DAT availability in the putamen measured with FE-PE2I (BP_ND_) versus FP-CIT (SUR) in patients and HC. DAT measurements are mean of the left and right putamen. Empty squares indicate HC that are not yet followed up. Filled squares indicate HC that are still healthy at follow-up after 2 years. Filled circles indicate patients that fulfilled clinical criteria for PS at inclusion and fulfilled clinical criteria for a specific parkinsonian syndrome at follow-up after 2 years. Empty circles indicate patients that fulfilled clinical criteria for PS at inclusion, not yet clinically followed up. Filled triangles indicate patients that fulfilled clinical criteria for PS at inclusion, but did not fulfil clinical criteria for a specific parkinsonian syndrome at follow-up after 2 years. DAT, dopamine transporter; *BP*_ND_, binding potential relative to non-displaceable binding; SUR, specific uptake ratio; FE-PE2I, [^18^F]FE-PE2I PET; FP-CIT, [^123^I]FP-CIT SPECT; PS, parkinsonian syndrome; HC, healthy controls

The cICC was high and significant between FP-CIT SUR and static FE-PE2I SUVR (caudate cICC = 0.870, putamen cICC = 0.796, and striatum cICC = 0.849, all *p* < 0.001) and even higher between FP-CIT SUR and dynamic FE-PE2I BP_ND_ (caudate cICC = 0.923, putamen cICC = 0.922, and striatum cICC = 0.946, all *p* < 0.001). A scatterplot of the mean DAT availability in the putamen (left and right hemispheres averaged) measured with FE-PE2I versus FP-CIT SPECT in patients and healthy controls is shown in Fig. 5.

### Correlation between DAT availability in the putamen and SN

**Fig. 6:**
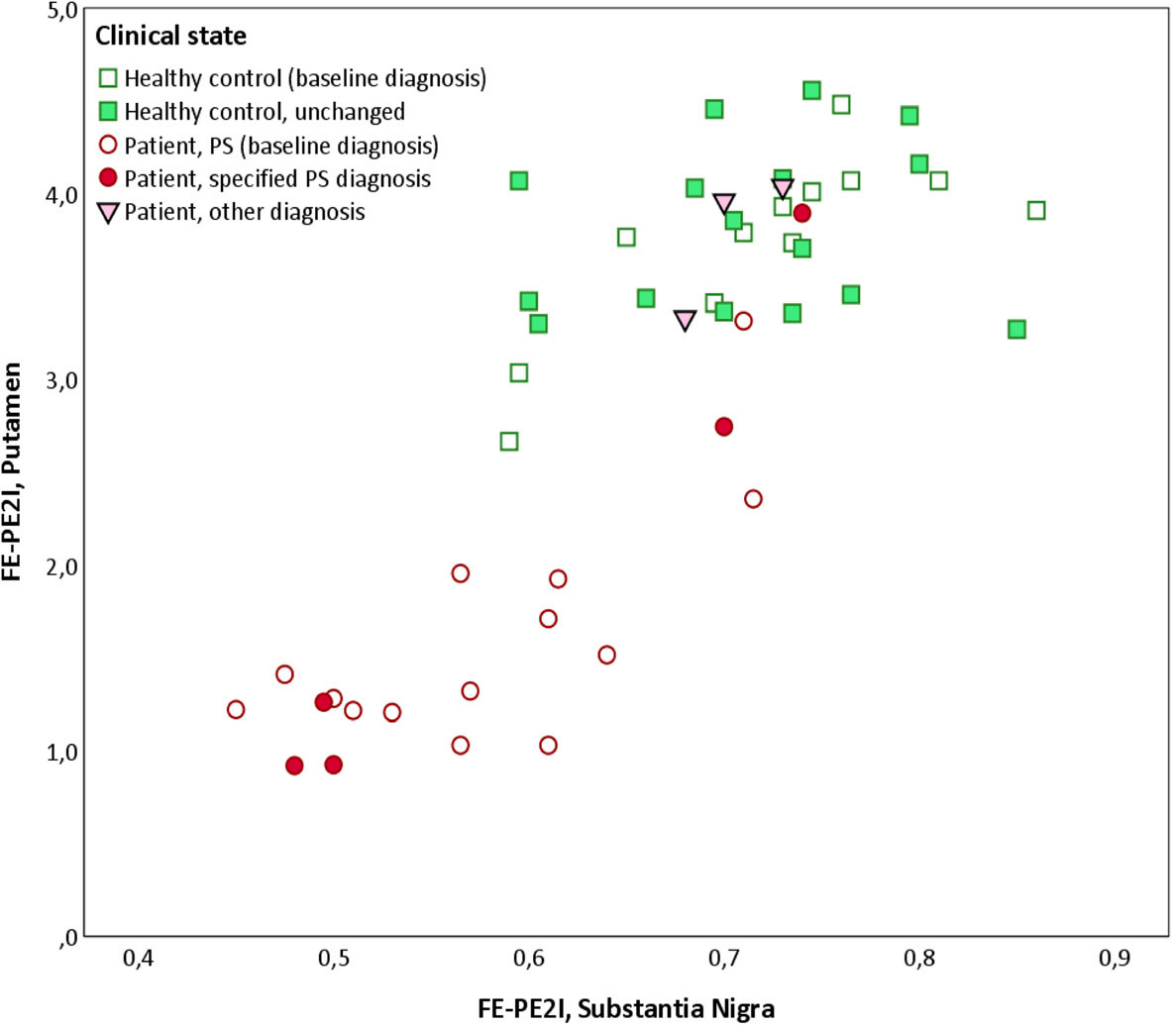
DAT availability in the putamen versus the substantia nigra. The relationship between the mean DAT availability in the putamen (left and right side averaged) and the SN (left and right side averaged), measured with FE-PE2I in HC and patients. Empty squares indicate HC that are not yet followed up. Filled squares indicate HC that are still healthy at follow-up after 2 years. Filled circles indicate patients that fulfilled clinical criteria for PS at inclusion and fulfilled clinical criteria for a specific parkinsonian syndrome at follow-up after 2 years. Empty circles indicate patients that fulfilled clinical criteria for PS at inclusion, not yet clinically followed up. Filled triangles indicate patients that fulfilled clinical criteria for PS at inclusion, but did not fulfil clinical criteria for a specific parkinsonian syndrome at follow-up after 2 years. HC, healthy controls; PS, parkinsonian syndrome; SN, substantia nigra; *BP*_ND_, binding potential relative to non-displaceable binding; FE-PE2I, [^18^F]FE-PE2I PET; FP-CIT, [^123^I]FP-CIT SPECT

In PS patients with reduced DAT in the putamen, there was a robust linear relationship between the DAT availability in the contralateral putamen and the DAT availability in the SN (average of right and left side) measured with FE-PE2I (*BP*_ND_: *R* = 0.690, *p* = 0.002; SUVR: *R* = 0.737, *p* = 0.001). In these patients, a clear correlation was also seen between contralateral putamen FP-CIT SUR and mean FE-PE2I DAT availability in the SN, measured both as *BP*_ND_ (*R* = 0.632, *p* = 0.007) and SUVR (*R* = 0.562, *p* = 0.019). In HC, there was a weaker correlation between the average DAT availability in the putamen and SN (FE-PE2I *BP*_ND_: *R* = 0.446, *p* = 0.017; FE-PE2I SUVR: *R* = 0.445, *p* = 0.018). In Fig. 6, the relationship between DAT availability in the putamen and the SN is illustrated. For simplicity, the *BP*_ND_ in the left and right hemispheres is averaged. This figure also shows that the patients with a normal DAT binding in the putamen also had DAT availability within normal limits in the SN. Even though the difference is statistically significant between patients and HC, there is clearly an interval, where the mean DAT activity in the SN is overlapping between groups.

### Correlation with clinical parameters

Clinical symptoms were lateralized to the right body half in 12 patients and the left side in eight patients. The clinically most affected body half corresponded with the lowest DAT binding in the contralateral hemisphere in the majority of the patients with both SPECT (90% in the caudate and putamen), with dynamic PET (85% in the caudate, 95% in the putamen) and static PET images (90% in the caudate, 85% in the putamen). In the SN, the contra-laterality correspondence was 60%.

In patients, there was no statistically significant correlation between measured DAT availability (with either of the imaging methods) and the duration of symptoms; neither was there any significant correlation with parkinsonism severity according to UPDRS-III, even though there was a trend towards a negative relationship. This did not change when controlling for age or sex, nor if the five patients with normal DAT were excluded from the analysis.

Olfaction was tested in 18/22 patients and in all healthy controls. In four patients, olfaction was normal (i.e., B-SIT score > 8 points) whereof one had CBS, one vascular parkinsonism, and one undeterminable tremor; the fourth patient, with essential tremor, was not tested. One patient with normal DAT availability (not yet clinically followed up) had impaired olfaction. Olfaction was abnormal in three HC (two of which have been confirmed still healthy after 2-year follow-up), and all of these had DAT availability well within the normal range. There were significant (*p* < 0.001) correlations between the B-SIT score and mean FP-CIT SUR in the caudate (*R* = 0.589), putamen (*R* = 0.729), and striatum (*R* = 0.710) as well as between B-SIT and mean FE-PE2I in the caudate (*BP*_ND_ *R* = 0.572, SUVR *R* = 0.455), putamen (*BP*_ND_ *R* = 0.616, SUVR *R* = 0.529), striatum (*BP*_ND_ *R* = 0.654, SUVR *R* = 0.539), and SN (*BP*_ND_ *R =* 0.447, SUVR *R* = 0.403).

## Discussion

Imaging of DAT availability with FE-PE2I PET/CT or FP-CIT SPECT/CT show excellent accuracy in distinction between HC and patients *with* de novo PS in an early clinical stage. A significant difference in DAT availability was measured between HC and PS. The ES was large and clinically relevant and was slightly higher for FE-PE2I than FP-CIT.

Both FE-PE2I and FP-CIT revealed normal striatal DAT availability in five patients who fulfilled the UKPDBB criteria for PS. These patients also displayed FE-PE2I binding in the SN within the range of HC. In three of these patients, the diagnosis at follow-up was essential tremor, undetermined tremor, and vascular parkinsonism, respectively. The fourth patient was clinically diagnosed with CBS at follow-up. It is known that CBS does not always show any signs of dopaminergic deficit in an early-stage disease [20, 21], and none of the imaging methods detected any DAT reduction in this patient, who had less than 1 year of symptoms’ duration at the time of imaging. The fifth patient with normal DAT availability has not yet been reassessed clinically.

According to the study protocol, all participants (including HC) will be clinically reassessed 24 months after inclusion, and future clinical follow-up will provide more clinical information in all participants and more specific clinical diagnoses for the patients.

A slight discrepancy between FE-PE2I and FP-CIT was represented in one HC who had relatively low DAT binding with FE-PE2I especially in the caudate, but normal FP-CIT SUR. When reviewing the MRI of this individual, very small lacunar infarcts were observed in the caudate and in the putamen, which may explain the lower and somewhat irregular DAT binding in PET. The higher spatial resolution observed with FE-PE2I PET imaging may be less permissive of such irregularities compared to SPECT imaging in which the spatial resolution is inferior and partial volume effects or smoothing may “compensate” for small focal lesions. This particular HC had midbrain DAT binding in the lower range among the HC, but intact olfactory function. It is unclear what this signifies, and this emphasizes the importance of clinical follow-up in both the healthy controls and the patients in this study. Some of the PS patients with significantly low FE-PE2I BP_ND_ in the putamen had DAT availability in the SN within the range of the HC, which is yet also of uncertain clinical significance. In addition, in Fig. 6, a slight overlap of measured DAT binding in the SN can be observed between HC and patients. The clinical follow-up of those PS and HC participants will probably help understand the relevance of these findings.

The FE-PE2I binding region in the midbrain was observed medial to and overlapping the part of the SN which has a low intensity on the T1 and SWAN MRI images. The low-intensity part of SN in these MRI sequences probably represents the SN pars reticulata [16]. However, the observed FE-PE2I binding region presumably originate from the DAT in the dopamine neuron-rich areas in the midbrain, e.g., SN pars compacta, which is a complex structure, difficult to visualize and delineate on conventional T1 and SWAN MRI sequences [22]. Further studies will elucidate these findings in more detail.

Age dependency has been reported for both men and women previously for both FP-CIT [23–26] and FE-PE2I [27]. In the present study, an age-related DAT decline in women but not in men was observed. This was previously reported in a different study using FP-CIT in healthy volunteers [23] within the same age range as that of the present study. It is interesting that the same phenomenon is observed also in the present study with both FP-CIT and FE-PE2I. Conceivably, the decline in women may possibly be accelerated in older age and therefore accentuated even in a small-sized group and constricted age range. In our study however, age differences did not have any relevant impact on the diagnostic accuracy. The lack of correlation between clinical severity, as measured with UPDRS-III, and DAT availability was also reported by Fazio et al. in a recently published study [3] and may be due to the early stage of disease in the patients and the small group size.

PET imaging with FE-PE2I allows for shorter interval between injection and imaging and shorter static imaging protocols (around 20 min, or less) than FP-CIT SPECT imaging (around 30 min). In particular, a short imaging protocol is an advantage for brain imaging of patients with movement disorders, since these patients often suffer from pain and stiff neck, which, apart from the disease-specific motor dysfunction, makes it difficult for many patients to lie still in prone position for longer times. In our study, the dynamic protocol is very long; however, static PET imaging may be accomplished in 20 min. In a recently published study [4], a static acquisition obtained in the time interval between 16.5 and 42 min, corresponding to the peak equilibrium of FE-PE2I, provided the best agreement with *BP*_ND_ with comparable effect size in differentiating PD patients from controls. In our study, we selected a 20-min static scan obtained at 50 min post injection, corresponding to the pseudoequilibrium of FE-PE2I, i.e., when the ratio between the target and the reference region becomes constant. This time window provided good diagnostic accuracy, with an ES between patients and HC slightly lower than the ES obtained using* BP*_ND_. We did not observe any systematic difference in ES between the 50–70-min static protocol and the previously reported time window [4] (data not shown). The image contrast observed in static images at 50–70 min post injection seems to be sufficient for visual interpretation in a clinical setting. The time window between 50 and 70 min also offers a practical solution similar to other frequently used PET imaging protocols, e.g., [^18^F]FDG of [^68^Ga]DOTATOC.

The primary objective of this study was to compare the performance of one established DAT imaging method, FP-CIT SPECT, with PET imaging using a new and more selective DAT radiopharmaceutical, FE-PE2I, in a clinically relevant setting, in which the clinical diagnosis is still unclear. In this situation, the diagnostic work-up of suspected PS can benefit from supporting evidence of reduced dopaminergic integrity by DAT imaging. A caveat with this study, as with imaging studies in PS in general, is the lack of a golden standard diagnosis (i.e., histopathological examination at autopsy) which is seldom an opportunity. A reliable clinical diagnosis does require a relatively long follow-up. The specific clinical diagnoses of the majority of the patients in this study remains to be confirmed. However, some of the participants do have a follow-up clinical diagnosis that corresponds well with the outcome of the SPECT and PET examinations.

The accessibility and costs of SPECT radiopharmaceuticals for DAT imaging vary between countries. The similar basic diagnostic results achieved with FE-PE2I PET compared to FP-CIT SPECT suggest that FE-PE2I PET is a valuable alternative DAT imaging method in cases of limited supply of DAT-SPECT radiopharmaceuticals. In the clinical setting, FE-PE2I PET has several advantages over FP-CIT SPECT: (i) superior DAT selectivity, (ii) shorter static imaging protocols without the need for pharmacological thyroid protection, (iii) better image resolution with PET, and (iv) possibility to obtain quantitative outcome measures with full dynamic PET acquisitions, when required. Additional studies across different centers are needed to further evaluate the advantages and other potential applications of FE-PE2I PET.

## Conclusions

This study confirms that DAT imaging with FE-PE2I PET exhibits excellent differentiation between subjects with and without dopaminergic deficit, offering a valuable supporting diagnostic tool for the evaluation of patients with newly onset clinical parkinsonism. FE-PE2I PET displays a high diagnostic correspondence with established imaging with FP-CIT SPECT, and either method may therefore be used with diagnostic confidence in the clinic. Further studies are warranted to assess the potential additional diagnostic information of imaging the DAT in the midbrain using FE-PE2I.

## Abbreviations

BP_ND_: Binding potential relative to non-displaceable binding
B-SIT: Brief Smell Identification Test™ (Sensonics International)
CBS: Corticobasal syndrome
CT: Computed tomography
DAT: Dopamine transporter
FE-PE2I: [^18^F]FE-PE2I, [^18^F]-(E)-*N*-(3-iodoprop-2-enyl)-2β-carbofluoroethoxy-3β-(4′-methyl-phenyl) nortropane
FP-CIT: [^123^I]FP-CIT, [^123^I]-ioflupane, DaTSCAN™ (GE Healthcare, B.V Eindhoven, NL)
H&Y: Hoehn and Yahr disease severity score
HC: Healthy control subjects
ICC: Intraclass correlation
LEHR: Low-energy high resolution
MRI: Magnetic resonance imaging
OSEM: Ordered subset expectation maximization
PET: Positron emission tomography
PS: Parkinsonian syndrome
SN: Substantia nigra
SPECT: Single-photon emission computed tomography
SUR: Specific uptake ratio
SUVR: Specific uptake value ratio
SWAN: Susceptibility-weighted angiography
UKPDBB: United Kingdom’s Parkinson’s Disease Society Brain Bank
UPDRS-III: Unified Parkinson’s Disease Rating Scale, motor part
VOI: Volume of interest
